# Editorial: Metabolic Adaptation of Muscle Tissue in Diseases Associated With Cachexia

**DOI:** 10.3389/fcell.2022.947902

**Published:** 2022-06-03

**Authors:** Federica Cirillo, Teresa A. Zimmers, Laura Mangiavini

**Affiliations:** ^1^ Laboratory of Stem Cells for Tissue Engineering, IRCCS Policlinico San Donato, San Donato Milanese, Italy; ^2^ Institute for Molecular and Translational Cardiology (IMTC), San Donato Milanese, Italy; ^3^ Department of Surgery, Indiana University School of Medicine, Indianapolis, IN, United States; ^4^ Indiana University Melvin and Bren Simon Comprehensive Cancer Center, Indianapolis, IN, United States; ^5^ Indiana Center of Musculoskeletal Health, Indianapolis, IN, United States; ^6^ Richard L. Roudebush Veterans Administration Medical Center, Indianapolis, IN, United States; ^7^ IRCCS Istituto Ortopedico Galeazzi, Milan, Italy; ^8^ Department of Biomedical Sciences for Health, University of Milan, Milan, Italy

**Keywords:** cachexia, skeletal muscle wasting, biomarker, metabolism, mitochondria

**FIGURE 1:**
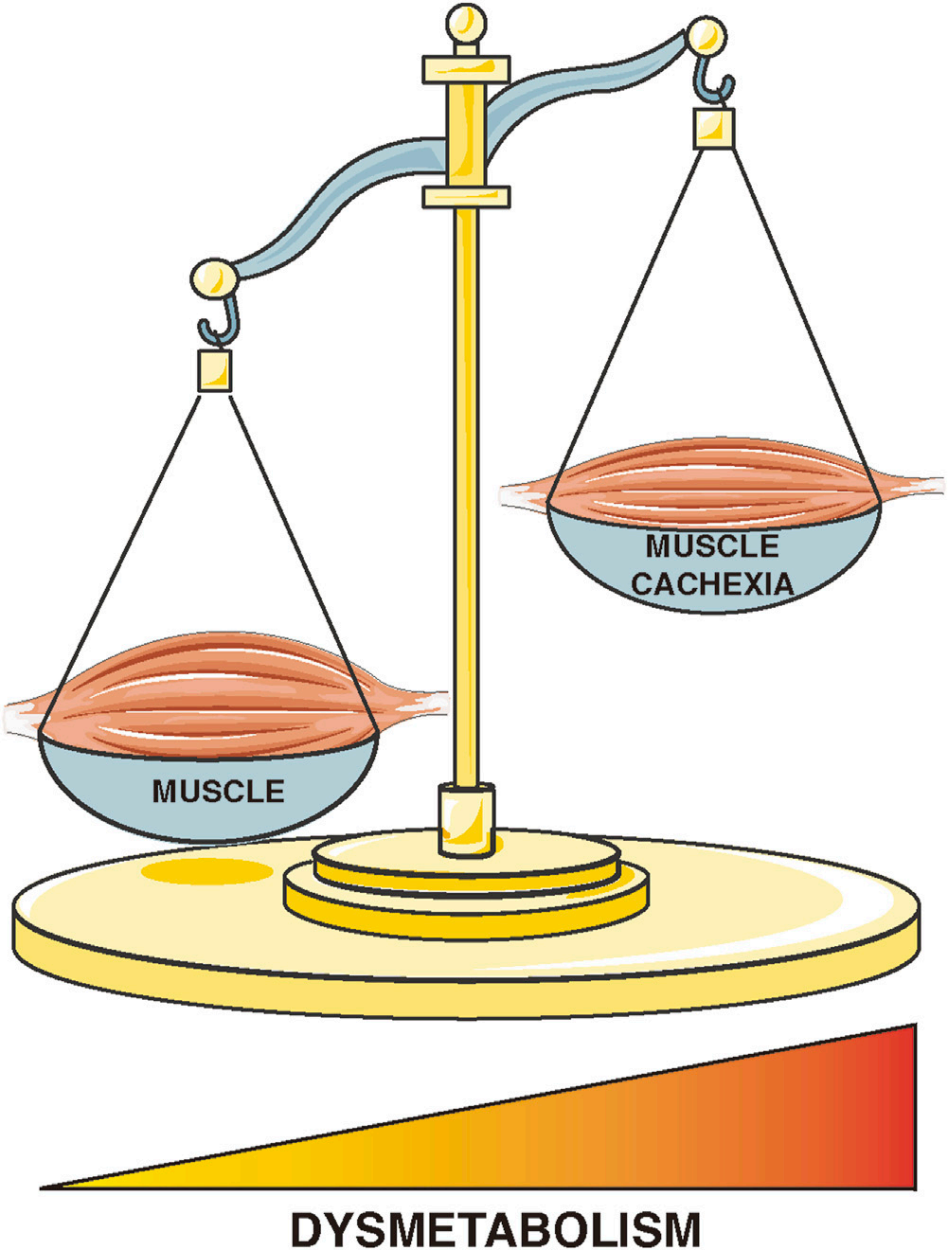
Schematic representation of changes in skeletal muscle metabolism in diseases associated with cachexia.

This Research Topic on skeletal muscle metabolism alterations in diseases associated with cachexia attempts to further elucidate how newly discovered molecular targets can influence muscle tissue structure and function. The skeletal apparatus, which accounts for approximately 30%–40% of total body weight (Kim et al., 2002), plays a critical physiological role in maintaining posture, controlling movement, altering thoracic volume for respiration, regulating temperature homeostasis, and cellular metabolism (Baskin et al., 2015; Frontera and Ochala, 2015; Rowland et al., 2015). Accelerated and excessive loss of skeletal muscle mass is a condition that occurs in several diseases, including cancer cachexia, which is closely correlated with a marked reduction in quality of life and shortened survival (Evans, 2010). In this context, cachexia has been recognized as a complex multifactorial metabolic syndrome characterized by glucose, lipid, and protein metabolism alterations, that contribute to the poor prognosis in affected patients (Rohm et al., 2019) (Figure 1). In recent years, the understanding of the molecular bases of these metabolic alterations has revealed novel targets for treating the syndrome. This generated an increasing number of new studies to further elucidate the molecular biomarkers of cachexia and to develop new therapeutic approaches that could counteract and reduce muscle wasting.

In this Research Topic, we collected four papers, three of which focus on cancer-derived cachexia and examine the role of several mitochondrial targets in influencing the metabolic profile during cancer progression.

In an elegant original article originating from patient samples and functionally interrogated in cultured murine myotubes, Mao et al. demonstrated that cachectic cancer patients exhibit reduced myofiber area, enlarged mitochondria with aberrant morphology and evidence of altered mitochondrial dynamics. Specifically, they observed upregulation of phosphorylated dynamin-related protein 1 (DRP1) at the Ser616 site, associated with markers of increased mitochondrial fission and consequent active fragmentation. The authors examined the relationship between DRP1, muscle degradation, and mitochondria using an *in vitro* model of cancer cachexia obtained by treating C2C12 myotubes with a conditioned C26 cell medium. They observed that inhibition of mitochondrial fission with Mdivi-1, a specific DRP1 inhibitor, counteracts muscle wasting by reducing protein degradation and improving mitochondrial function. While DRP1 regulates mitochondrial fission under normal physiological conditions, its overexpression in cancer patients with cachexia leads to promote myocellular mitochondrial dysfunction and myotube wasting.

Mitochondrial alterations, as a target for cachexia therapy, are also the main object of original article by Pin et al. The authors examined the effects of Mitoquinone Q (MitoQ), one of the most widely used antioxidants for mitochondria, on skeletal muscle wasting and metabolism *in vitro* and *in vivo*. Specifically, they found that MitoQ protects myotubes from atrophy *in vitro* by preventing increases in Atrogin-1 and Murf1 gene expression. In addition, they described MitoQ as a tool to prevent mitochondrial changes and improve the cachectic phenotype in C26 tumor-bearing mice. Specifically, MitoQ administration partially corrected skeletal muscle atrophy in male CD2F1 mice inoculated with C26 tumor cells. The authors demonstrated that MitoQ is an effective approach to improve mitochondrial function and metabolism by promoting a shift in fiber composition from glycolytic to oxidative, confirming the anticachectic properties of MitoQ *in vivo*. The authors suggest MitoQ could be administered concomitantly with chemotherapeutic agents routinely used in the clinic to counteract cancer-induced skeletal muscle atrophy. Whether MitoQ would be protective against chemotherapy-induced cachexia in the context of cancer or affect tumor response to chemotherapy remains to be determined in pre-clinical models.

The discovery of novel metabolic biomarkers involved in the onset and progression of cachexia is necessary to develop new therapies to treat patients and improve disease outcomes. In this context, O’Connell et al. have developed an original article in an experimental model, focused on identifying molecular targets of cancer cachexia in the early stages with the goal of preventing refractory phase, which is characterized by patients becoming unresponsive to therapies and exhibiting uncontrolled weight loss. The unique feature of this study is the multiplatform strategy used (NMR, MS, and NMR-based lipoprotein platforms) to profile progressive changes in the metabolome across the time course in C26 tumor-bearing mice. The authors found that the tumor progression induces significant differences in circulating amino acids, acylcarnitines, and lipoproteins prior to evident weight loss. These findings suggest that for patients, specific plasma metabolite biomarkers could serve as diagnostic predictors of cancer cachexia development or progression. Such a validated biomarker panel in patients would greatly facilitate drug development by identifying patients at risk of developing cachexia and monitoring response to therapy.

The original article by Wen et al. addresses potential mechanisms driving the loss of skeletal muscle tissue in patients exposed to long-term controlled mechanical ventilation (CMV) in Intensive Care Units (ICUs). Specifically, CMV results in severe cachectic phenotype with impaired strength and movement, as well as alterations in other important skeletal muscle functions such as secretion of hormones, formation of metabolites, and reserve of amino acids. The authors conducted an *in vivo* study using Sprague-Dawley rats exposed to CMV for 5 days and then performed a metabolomic analysis of two respiratory muscles (diaphragm and intercostal muscle) and lung tissue. The authors showed a dramatic change in the metabolomic profile of lipids (Acyl Carnitine) and amino acids (Leucine, Isoleucine and Valine) with a specific signature for each tissue associated with increased protein breakdown in the two respiratory muscles, active inflammation in all tissues, attenuated energy production in the muscles, and enhance energy production in the lungs. This provides a pre-clinical basis for future studies in patients and for the ultimate discovery of biomarkers for early diagnosis to prevent the risk of secondary pulmonary complications and mortality.

In conclusion, the articles published in this Research Topic illustrate novel insights and highlight the role of skeletal muscle metabolism as a potential candidate for exploring innovative therapeutic approaches for the treatment of diseases associated with cachexia.
